# The Genetic Analysis Workshop 16 Problem 3: simulation of heritable longitudinal cardiovascular phenotypes based on actual genome-wide single-nucleotide polymorphisms in the Framingham Heart Study

**DOI:** 10.1186/1753-6561-3-s7-s4

**Published:** 2009-12-15

**Authors:** Aldi T Kraja, Robert Culverhouse, E Warwick Daw, Jun Wu, Andrew Van Brunt, Michael A Province, Ingrid B Borecki

**Affiliations:** 1 Division of Statistical Genomics, Washington University School of Medicine, 4444 Forest Park Boulevard, Campus Box 8506, St. Louis, Missouri 63108, USA; 2 Division of General Medical Sciences, Washington University School of Medicine, 660 South Euclid Avenue, Box 8005, St. Louis, Missouri 63110, USA

## Abstract

The Genetic Analysis Workshop (GAW) 16 Problem 3 comprises simulated phenotypes emulating the lipid domain and its contribution to cardiovascular disease risk. For each replication there were 6,476 subjects in families from the Framingham Heart Study (FHS), with their actual genotypes for Affymetrix 550 k single-nucleotide polymorphisms (SNPs) and simulated phenotypes. Phenotypes are simulated at three visits, 10 years apart. There are up to 6 "major" genes influencing variation in high- and low-density lipoprotein cholesterol (HDL, LDL), and triglycerides (TG), and 1,000 "polygenes" simulated for each trait. Some polygenes have pleiotropic effects. The locus-specific heritabilities of the major genes range from 0.1 to 1.0%, under additive, dominant, or overdominant modes of inheritance. The locus-specific effects of the polygenes ranged from 0.002 to 0.15%, with effect sizes selected from negative exponential distributions. All polygenes act independently and have additive effects. Individuals in the LDL upper tail were designated medicated. Subjects medicated increased across visits at 2%, 5%, and 15%. Coronary artery calcification (CAC) was simulated using age, lipid levels, and CAC-specific polymorphisms. The risk of myocardial infarction before each visit was determined by CAC and its interactions with smoking and two genetic loci. Smoking was simulated to be commensurate with rates reported by the Centers for Disease Control. Two hundred replications were simulated.

## Background

The Framingham Heart Study (FHS) is a rich platform for the study of cardiovascular disease and the application of novel, imaginative analytic strategies. For Genetic Analysis Workshop (GAW) 16, we use a semi-simulated approach using actual genotypes from the 500 k Affymetrix platform and the 50 k candidate gene chip and building phenotypes on the observed genetic variation. Because blood lipid levels are a major risk factor in the development of cardiovascular disease [1], we modeled disease risk on the lipid pathway, including both genetic and environmental determinants. The FHS has reported that long-term averages of low-density lipoprotein (LDL), high-density lipoprotein (HDL), and triglyceride (TG) levels were highly heritable (0.66, 0.69, and 0.58, respectively) [2]. Several familial studies also have reported heritabilities for LDL of 0.50, HDL of 0.54, and TG of 0.39 [3]. Dyslipidemia, as a fundamental component of the atherosclerotic process, is a medically correctable risk factor with established efficacious treatments for reducing risk of coronary heart disease [4]. Thus, we included in our simulation the use and effects of dyslipidemic medications, which have an important role in shaping lipid profiles. This simulation builds in the long tradition of previous simulations for Genetic Analysis Workshops [5,6].

## Methods

The FHS pedigrees, distributed as GAW16 Problem 2, formed the basis of our simulation [7]. In total, there were 6,476 subjects who had genotypes and simulated phenotypes. After the simulations began, additional FHS subjects provided broad consent for data sharing; these additional subjects were not included in the simulations. To ensure comparable data to that which was simulated, we provided a file that defined precisely which subjects were included and their relationships within families. The ~550 k measured single-nucleotide polymorphism (SNP) genotypes, distributed for GAW16 Problem 2 from both the genome-wide scan and the additional candidate gene platform (GeneChip^® ^Human Mapping 500 k Array Set (Nsp and Sty), and the 50 k Human Gene Focused Panel) comprised the genotypes for GAW16 Problem 3. Novel fictitious phenotypes were simulated for subjects.

Although family members of the FHS attended various exams at different times, depending on the generation, we modeled our study as if all subjects were recruited at one time, calculated the family member's relative ages at one particular exam, and then assigned a simulated age for everyone at three time points, with 10-year intervals. The mean age in years (range) for the simulation, by generation and visit, is shown in Table 1.

**Table 1:** Mean ages of the simulated data (mean, minimum, and maximum age in years)

	Mean age (minimum, maximum)		
	
Generation	Visit 1	Visit 2	Visit 3
1	66 (54, 80)	76 (64, 90)	86 (74, 100)
2	56 (20, 80)	66 (30, 90)	76 (40, 100)
3	33 (19, 70)	43 (29,80)	53 (39,90)
Overall	43 (19, 80)	53 (29, 90)	63 (39, 100)

**Figure 1:**
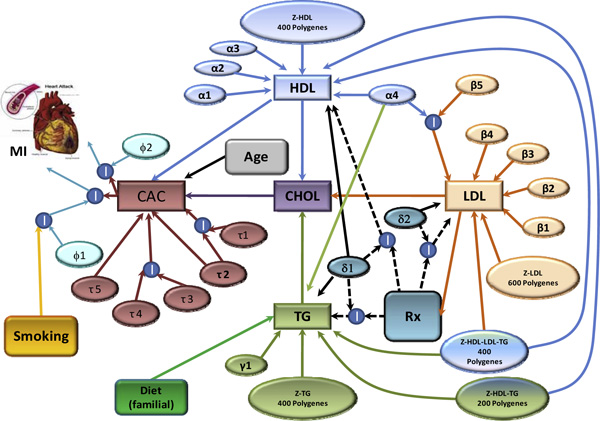
**The Genetic Analysis Workshop 16 Problem 3 diagram**. Figure 1 shows simulated phenotypes emulating the lipid domain (HDL, LDL, TG, and CHOL) and its contribution to cardiovascular disease risk (CAC and MI). Simulated major genes are symbolized with Greek letters. There are 1,000 polygenes for each trait HDL, LDL, and TG, several of them with pleiotropic effects. Continued lines and arrows show causality/interaction (I); dashed lines show pharmacogenetic effects only for subjects treated with medication, where response was dependent on the subjects' genotypes. Environmental factors such as diet, smoking, and medication were modeled in the simulation.

The simulation model is depicted in Figure 1. There are up to six "major" genes for the lipid phenotypes HDL, LDL, and TG, and 1,000 polygenes for each trait. Several polygenes have pleiotropic effects (i.e., several of these polygenes affect two or three or trait combinations simultaneously). The identity and effects of the major genes are documented in Table 2. The locus-specific heritabilities of the major genes range from 0.1-1.0% under additive (AA:AB:BB, 0:0.5:1), dominant (AA:AB:BB, 1:1:0), or overdominant (AA:AB:BB, 0:1:0; heterozygotes show higher effect than the two homozygotes) modes of inheritance, with minor allele frequencies at least 5%, with one exception (*β*4), for which the minor allele frequency was 1%. We simulated an overdominant effect (*γ*1) because there appears to be evidence supporting this possibility and this mode of inheritance is rarely, if ever, modeled. The gene *α*4 is pleiotropic for HDL and TG and interacts with *β*5 in determining LDL (Figure 1). The interaction accounts for 0.7% of the trait variance, and *β*5 has no marginal effect on any phenotype. The locus-specific effects of the polygenes were on average an order of magnitude smaller, ranging from 0.002-0.15%, with effect sizes extracted from negative exponential distributions. All polygenes act independently and have additive effects. HDL, TG, and LDL share 40% of their polygenes in common, and HDL and TG share an additional 20%. The specific identities of the polygenes, their locations, and their generating effect sizes are provided in the Additional Files 1, 2, 3 corresponding to HDL, LDL, and TG. A group of 39 polygenes influencing HDL were clustered within 0.5 Mb on chromosome 11; otherwise, the polygenes for each trait are randomly distributed throughout the genome. The overall effect of each trait-specific polygenic component was scaled to achieve the target total trait heritabilities of 60%, 55%, and 40% for HDL, LDL, and TG, respectively. The remaining variance is uncorrelated among family members, with the exception of a simulated dietary effect (variable: diet) on TG levels that accounts for a correlation of 0.05 among family members, regardless of their coefficient of relationship. The phenotypes generated from this genetic model were scaled to the empirically derived means and variances for the actual HDL, LDL, and TG traits within 13 age strata (in 5-year intervals) and sex as follows:

**Table 2:** Summary characteristics of the major genes and polygenes for traits HDL, LDL, and TG^a^

**No**.	Trait	Chr	Symbol^b^	Gene	SNP	Role	Total	*h* ^2^	Model^c^	MAF	*h*^2 ^of polygenes
1	HDL	9q31.1	*α*_1_	*ABCA1*	rs10820738	Intron/Exon boundary		0.010	DOM	6.7	
2	HDL	19q13.2	*α*_2_	*CYP2B7P1*	rs8103444	Exon/Intron boundary		0.002	ADD	24.4	
3	HDL	15q21	*α*_3_	*LIPC*	rs8035006	Intron		0.003	ADD	32.5	
4	HDL	8p22	*α*_4_	*LPL*	rs3200218	Exon downstream		0.003	DOM	21.7	
5	HDL	19q13.2	*δ*_1_	*CYP2B6*	rs8192719	Exon/Intron boundary (Rx)		0.003	ADD (up 10%)^d^	24.9	
											
							Total	0.021			
6	HDL	1,000 SNPs	[apoly]					0.58	ADD	≠	min = 0.0003; max = 0.0015; avg = 0.00058
											
							Total	0.60			
1	LDL	4p16.3	*β*_1_	*LRPAP1*	rs7672287	Intron		0.003	ADD	22.2	
2	LDL	12q13	*β*_2_	*LRP1*	rs1466535	Intron		0.002	ADD	31.6	
3	LDL	11q13.4	*β*_3_	*LRP5*	rs901824	Intron/Exon boundary		0.001	ADD	10.3	
4	LDL	chrom1	*β*_4_		rs10910457			0.005	ADD	1.0	
5	LDL	8p22 × 4q24	*β*_5_	*LPL × NFKB1*	rs4648068	Intron		0.007	INT	31.0	
6	LDL	22q12.2	*δ*_2_	*SLC5A4*	rs2294207	Intron	(Rx)	0.010	ADD (down 30%)	25.7	
											
							Total	0.028			
7	LDL	1,000 SNPs	[bpoly]					0.52	ADD	≠	min = 0.0003; max = 0.00128; avg = 0.00052
											
							Total	0.55			
1	TG	11q23	*γ*_1_	*APOA5*	rs603446	Downstream		0.003	OVERD	43.3	
2	TG	8p22	*α*_4_	*LPL*	rs3200218	Exon downstream		0.004	ADD	21.7	
3	TG	19q13.2	*δ*_1_	*CYP2B6*	rs8192719	Exon/Intron boundary (Rx)		0.003	ADD (down 15%)	24.9	
4	TG		diet					0.01	Familial		
											
							Total	0.020			
5	TG	1,000 SNPs	[gpoly]					0.38	ADD	≠	min = 0.0002; max = 0.0009; avg = 0.00038
											
							Total	0.40			

^a^Abbreviations: MAF, Minor allele frequency; min, minimum; max, maximum; avg, average; *h*^2^, heritability; [apoly], a vector of 1,000 polygenes that contribute to HDL; [bpoly], a vector of 1,000 polygenes that contribute to LDL; [gpoly], a vector of 1,000 polygenes that contribute to TG.

^b^Symbol represents a locus/a vector of polygenes marked by SNPs and corresponding genes, which can be introns/exons, or a diet effect.

^c^Effects of genes were simulated based on ADDitive (0, 0.5, 1), DOMinant (1, 1, 0), OVERDominant (0, 1, 0), and INTeraction genetic models.

^d^Medication treatment lowered LDL on a varying percentage of participants dependent on visits: 2% of subjects in Visit 1, 5% of subjects in Visit 2, and 15% of subjects in Visit 3. Medication treatment increased HDL 10%, lowered LDL 30%, and lowered TG 15% for the treated subjects and in accordance with specific types of genotypes, shown in the "Model" column. For HDL on chromosome 11, 39 SNPs were simulated as a block of polygenes, starting at 110 Mbp and ending at 134 Mbp, with approximately an average equidistance of 0.5 Mbp.

where, for example, _(HDL|age 5 year interval, sex) _represents the mean of HDL in FHS, given a 5-year age interval and sex; _(HDL|age 5 year interval, sex) _is standard deviation of HDL in FHS, given a 5-year age interval and sex; *h*_*α*1 _is the square root of simulated heritability for the *α*_1 _SNP (as described in Table 2); *a*_*α*1 _is a simulated effect that reflects in part the penetrance of the *α*_1 _SNP; *sign *is a random integer number that takes values (-1) or (+1) with the purpose of randomly changing the contribution direction of polygenes; *apoly*_κ _represents an instance of each of the 1,000 SNPs effects (*k *= 1 to 1,000), selected as polygenes for HDL; *hapoly*_κ _is an instance of the of square roots of heritabilities for 1,000 SNPs selected as polygenes for HDL; *a*_*ε *_represents the environmental effect that contributes to HDL; and *h*_*ε *_is the square root of HDL variance explained by environmental causes.

As individuals progressed to the next visit 10 years later, their phenotypes were scaled by the appropriate age-sex means and variance, but there are no genes governing longitudinal trends *per se*. Instead, we simulated the complicating effects of medication. The simulated value for LDL at each visit for each subject was checked, and individuals in the upper tail of the distribution were simulated as medicated. The proportion of subjects that are medicated increased across visits to comprise 2%, 5%, and 15% of the subjects in Visits 1, 2, and 3, respectively. These proportions were estimated from the FHS data, and reflected the secular increase in the proportion of individuals being treated for elevated cholesterol levels. The response to treatment is governed by two loci (*δ*1 and *δ*2) as pharmacogenetic processes. The *δ*1 variant has a marginal effect on both HDL and TG levels via additive effects but also, individuals that are homozygous for the minor allele are non-responders to the treatment. Responders (homozygotes and heterozygotes for the major allele) exhibit a 10% increase in their HDL levels and a 15% decrease in TG levels. Similarly, *δ*2 is a variant with an additive marginal effect on LDL, and homozygotes for the minor allele are non-responders to treatment. Responders exhibit a 30% decrease in LDL levels. Total cholesterol (CHOL) level is calculated as 0.8*(HDL + LDL + TG/5), and has no independent genetic effects except those influencing the component phenotypes.

Coronary artery calcification (CAC) was simulated as a quantitative phenotype that takes many years to develop. For this reason, CAC was modeled in two stages. First, age-independent CAC (CAC_AI_) was modeled as a function of total CHOL, HDL, and five other genes (*τ*1-*τ*5) having direct effects on its development. CAC_AI _was simulated under the model

where **ME **is a joint genetic effect from an epistatic interaction between *τ*1 and *τ*2, the effect of *τ*1 is purely epistatic (i.e., *τ*1 displays only a minimal main effect) while *τ*2 displays an additional measurable additive main effect; **PE **is the joint effect from *τ*3 and *τ*4, a pair of purely epistatic SNPs, each with no main effect; **Het **is an effect from *τ*5, a SNP that displays heterosis (over-dominance); and *ε *is the residual variation not explained by the factors mentioned above. The term *ε*, 300 times a random draw from a normal distribution with mean 0 and variance 1 (300 × *N*(0,1)), represents the sum of normal deviations from the mean of each of the modeled genetic effects and "noise" from unmeasured environmental and genetic effects. Because CAC cannot be negative, CAC_AI _= 0 if the generated value was negative. The models for the effects on CAC_AI _due to the **ME **and **PE **genotypes are illustrated in Tables 3 and 4. The minor allele frequency (MAF) for each of the four SNPs *τ*1-*τ*4 is ~0.5. SNP *τ*5, which determines the **Het **effect, has a MAF of 0.2. SNP *τ*5 genotype 1/1 (common homozygote) increases CAC_AI _on average by 25, genotype 1/3 decreases CAC_AI _by 100, and genotype 3/3 increases CAC_AI _by 400. CAC is derived from CAC_AI _by using a piecewise linear age adjustment: subjects under 20 years have not developed measurable levels of CAC, CAC buildup is linear from the ages of 20 to 60, and for subjects older than 60, CAC = CAC_AI_. Table 5 lists estimates of the proportion of the variability of CAC attributable to each of the genetic factors averaged over the 200 replicate datasets.

**Table 3:** Mean effects of ME (*τ*1 and *τ*2) on CAC_AI_

		***τ*2**			
		**2/2**	**2/4**	**4/4**	**marginal affects**
	
*τ***1**	**2/2**	- 250	0	250	0
	
	**2/4**	150	0	- 150	0
	
	**4/4**	- 250	0	250	0
	
	**marginal affects**	- 100	0	100	

**Table 4:** Mean effects of PE (*τ*3 and *τ*4) on CAC_AI_

		***τ*4**		
		**1/1**	**1/2**	**2/2**
		
*τ***3**	**2/2**	200	- 200	200
		
	**2/4**	- 200	200	- 200
		
	**4/4**	200	- 200	200

**Table 5:** Proportion of explained variability for the genetic factors contributing to CAC^a ^(by visit)

		Factor		L1^b^		L2	
				
Factor	Visit	Mean	(Min - Max)	Mean	(Min - Max)	Mean	(Min - Max)
ME	1	0.0053	0.0037 - 0.0065	0.00002	0.0^c^ - 0.00012	0.00030	0.00008 - 0.00063
	2	0.0092	0.0075 - 0.0112	0.00003	0.0 - 0.00013	0.00055	0.00015 - 0.00093
	3	0.0115	0.0091 - 0.0137	0.00003	0.0 - 0.00019	0.00066	0.00027 - 0.00127
							
PE	1	0.0091	0.0076 - 0.0115	0.00004	0.0 - 0.00020	0.00003	0.0 - 0.00014
	2	0.0176	0.0152 - 0.0212	0.00004	0.0 - 0.00017	0.00004	0.0 - 0.00019
	3	0.0226	0.0191 - 0.0266	0.00004	0.0 - 0.00021	0.00004	0.0 - 0.00016
							
Het	1	0.0021	0.0012 - 0.0032				
	2	0.0045	0.0032 - 0.0060				
	3	0.0062	0.0045 - 0.0080				

^a^Models included the genetic factors age, sex, CHOL, and HDL.

^b^Columns L1 and L2 indicate the effect of each of the epistatic loci when analyzed without its mate.

^c^0.0 indicates *R*^2^<10^-5^

Whether a subject smoked during the period before a visit influenced the risk of a myocardial infarction (MI). At first visit, men had a 27% chance to be smokers and women had a 23% chance. Each smoker had an 8% chance of permanently quitting smoking before each subsequent visit. The resulting smoking rates are commensurate with rates reported by the Centers for Disease Control for 1998. The risk of an MI before each visit is determined by CAC and its interactions with smoking and two genetic loci, *φ*1 and *φ*2. No MIs were fatal in our data. Smoking and *φ*1 have an interactive effect on risk of MI. The effect of smoking is to constrict blood vessels, thus increasing the risk that CAC will lead to an MI. The risk of MI for a smoker with the most common *φ*1 genotype (3/3) is the same as that of an equivalent non-smoker whose CAC is 10% higher. The risk of MI for a smoker with either of the other *φ*1 genotypes is the same as that of a non-smoker whose CAC is 40% higher. The *φ*1 genotype has no effect on risk of MI in non-smokers. Carrying the most common *φ*2 genotype (3/3) has the same effect on risk of MI as reducing CAC by 5%. The effect of any other genotype is the same as increasing CAC by 5%. The final model for MI risk is

where ∂_*smoke *_is the joint effect of smoking and *φ*1 (0 if a non-smoker, 0.1 if a smoker with genotype 3/3 at *φ*1, 0.4 if a smoker with another genotype); and the value of the *event *variable is -0.05 if the *φ*2 genotype is 3/3 and 0.05 otherwise. The MAFs for *φ*1 and *φ*2 are ~0.3. MI_risk was calculated for each visit and a draw from a uniform distribution determined whether the risk resulted in an MI. The SNPs for CAC and MI event were chosen from the 50 k SNPs in the Gene Focused Panel based on desired MAF, completeness of genotyping, and lack of linkage disequilibrium between the SNPs. The specific identities of the SNPs *τ*1-*τ*5, *φ*1 and *φ*2, and their chromosomes are listed in Table 6.

**Table 6:** SNPs contributing to CAC and MI event

Trait	Factor	RS number	MAF	Chr
CAC	*τ*1	rs6743961	0.4997	2
	*τ*2	rs17714718	0.5000	19
	*τ*3	rs1894638	0.4990	6
	*τ*4	rs1919811	0.4994	7
	*τ*5	rs213952	0.2000	7
				
MI	*φ*1	rs12565497	0.3001	1
	*φ*2	rs11927551	0.2999	3

## Results and discussion

The phenotypic simulated files are named simphen#.txt, where # stands for a number from 1-200, representing the replication number. The simulated data are archived in the dbGAP of the National Center for Biotechnology Information under the name "GAW16 Framingham and Simulated Data" [8]. The 200 replications of the data include the indexing variable "shareid" that matches exactly with the same shareid of the Framingham Heart Study and can be used to merge the simulated phenotypic data with the FHS genotypic data. The phenotypic variables provided are sex, simage (simulation age), diet, rx (antihyperlipidemic medication use), LDL, HDL, TG, CHOL, CAC, SMOKE, and MIevent, each associated with a number (1, 2, or 3) to identify respectively variables that were simulated for Visit 1, Visit 2, or Visit 3.

We tested all the simulated traits and causative SNP heritabilities as well as the respective association models. Analyzing and interpreting data obtained as part of a genome-wide association study presents numerous challenges, as well as the promise of improved understanding of the genetic factors influencing complex traits. For validation and a detailed analysis of the simulated model see the Online Supplemental Materials for GAW16 [9]. Many genome-wide association studies have been published recently, and many more are being carried out on virtually every conceivable phenotype of biomedical or public health importance. While the rate of development of genetic technologies has propelled us to this point, development and evaluation of statistical and analytic techniques is still underway, with many issues not yet satisfactorily resolved. Nonetheless, important discoveries have been reported. We hope that the simulated GAW16 Problem 3 provides data with which investigators can test the strengths and limitations of their statistical analytic approaches and software.

## List of abbreviations used

CAC: Carotid arterial calcification; CHOL: Cholesterol; FHS: Framingham Heart Study; GAW: Genetic Analysis Workshop; HDL: High-density lipoprotein; LDL: Low-density lipoprotein; MAF: Minor allele frequency; MI: Myocardial infarction; SNP: Single-nucleotide polymorphism; TG: Triglyceride.

## Competing interests

The authors declare that they have no competing interests.

## Authors' contributions

All the authors contributed equally.

## Supplementary Material

Additional file 1 Heritability targets and other characteristics for each polygene affecting HDL. Click here for file

Additional file 2 Heritability targets and other characteristics for each polygene affecting LDL. Click here for file

Additional file 3 Heritability targets and other characteristics for each polygene affecting TG. Click here for file
